# Mechanism of primitive duct formation in the pancreas and submandibular glands: a role for SDF-1

**DOI:** 10.1186/1471-213X-9-66

**Published:** 2009-12-14

**Authors:** Anne-Christine Hick, Jonathan M van Eyll, Sabine Cordi, Céline Forez, Lara Passante, Hiroshi Kohara, Takashi Nagasawa, Pierre Vanderhaeghen, Pierre J Courtoy, Guy G Rousseau, Frédéric P Lemaigre, Christophe E Pierreux

**Affiliations:** 1 Université catholique de Louvain, de Duve Institute, 75 Avenue Hippocrate, B-1200 Brussels, Belgium; 2 Institut de Recherche Interdisciplinaire en Biologie Humaine et Moléculaire, Free University of Brussels, 808 Route de Lennik, B-1070 Brussels, Belgium; 3 Institute for Frontier Medical Science, 53 Kawahara-cho, Shogoin, Sakyo-ku, Kyoto 606-8507 Japan

## Abstract

**Background:**

The exocrine pancreas is composed of a branched network of ducts connected to acini. They are lined by a monolayered epithelium that derives from the endoderm and is surrounded by mesoderm-derived mesenchyme. The morphogenic mechanisms by which the ductal network is established as well as the signaling pathways involved in this process are poorly understood.

**Results:**

By morphological analyzis of wild-type and mutant mouse embryos and using cultured embryonic explants we investigated how epithelial morphogenesis takes place and is regulated by chemokine signaling. Pancreas ontogenesis displayed a sequence of two opposite epithelial transitions. During the first transition, the monolayered and polarized endodermal cells give rise to tissue buds composed of a mass of non polarized epithelial cells. During the second transition the buds reorganize into branched and polarized epithelial monolayers that further differentiate into tubulo-acinar glands. We found that the second epithelial transition is controlled by the chemokine Stromal cell-Derived Factor (SDF)-1. The latter is expressed by the mesenchyme, whereas its receptor CXCR4 is expressed by the epithelium. Reorganization of cultured pancreatic buds into monolayered epithelia was blocked in the presence of AMD3100, a SDF-1 antagonist. Analyzis of *sdf1 *and *cxcr4 *knockout embryos at the stage of the second epithelial transition revealed transient defective morphogenesis of the ventral and dorsal pancreas. Reorganization of a globular mass of epithelial cells in polarized monolayers is also observed during submandibular glands development. We found that SDF-1 and CXCR4 are expressed in this organ and that AMD3100 treatment of submandibular gland explants blocks its branching morphogenesis.

**Conclusion:**

In conclusion, our data show that the primitive pancreatic ductal network, which is lined by a monolayered and polarized epithelium, forms by remodeling of a globular mass of non polarized epithelial cells. Our data also suggest that SDF-1 controls the branching morphogenesis of several exocrine tissues.

## Background

Branching morphogenesis is a process that allows the formation of a branched network of tubes, as exemplified by the airways of the lung or the excretory ducts of the pancreas and salivary glands [1,2]. During branching morphogenesis, the epithelial cells interact with the surrounding mesenchyme and organize into polarized monolayers with their apical pole facing the tube lumen [3,4]. How this process takes place and is regulated in exocrine tissues such as the pancreas and salivary glands remains poorly understood.

In the mouse, the pancreas originates from a pre-patterned endodermal epithelium located in a caudal region of the foregut that is to become the duodenum. Between embryonic days (e) 8.5 and e9.5, two outgrowths develop from the dorsal and ventral sides of this endodermal region, and form epithelial buds surrounded by mesenchyme. From e9.5-e10.5 onwards, the pancreatic bud cells proliferate, differentiate and undergo extensive morphogenesis to generate ductal structures called primitive ducts. The latter then expand, and give rise to the endocrine islets of Langerhans and to a branched ductal network that drains the secretions of the exocrine acini [5-10]. The submandibular glands (SMG) also derive from the foregut endoderm. Their development starts around e11.5 by formation of two epithelial thickenings beneath the tongue. These thickenings protrude into the underlying mesenchyme. Around e13.5, small clefts appear at the periphery of the budding epithelial mass, and after continuous proliferation and repetitive clefting, a tree-like network of ducts whose branches end in acini is generated [11,12].

Regulation of epithelial morphogenesis in the pancreas and SMG is controlled by the surrounding mesenchyme [13,14]. Moreover, gene inactivation studies and *ex vivo *culture experiments have identified several signaling molecules that regulate SMG branching morphogenesis [15-19]. In the developing pancreas, gene inactivation studies inhibiting FGF10, EGF, or Rbpj expression revealed impaired branching morphogenesis. However, these studies focused on the role of the signaling molecules on pancreatic cell differentiation and not on the mechanisms of branching [20-23].

Stromal cell-Derived Factor-1 (SDF-1, also called CXCL12 or PBSF) is a secreted protein of the α-chemokine family, and a potent chemoattractant for many cell types [24-26]. Whereas SDF-1 is the sole ligand for the chemokine CXC-motif receptor 4 (CXCR4), CXCR7 can bind SDF-1 and CXCL11/I-TAC [27]. *Sdf1 *and *cxcr4 *knockout mice die perinatally and display profound defects in the hematopoietic and nervous system [28-32], whereas *cxcr7 *knockout embryos die at birth due to defects in heart formation [33]. No role has been ascribed to SDF-1/CXCR4 signaling in the SMG. In contrast, two functions for SDF-1 signaling in adult pancreas have been proposed. One day before birth, when pancreatic cells still differentiate and extensive islet neogenesis occurs, CXCR4 is expressed in endocrine cells and in some ductal cells, whereas SDF-1 is only found in endocrine cells [34]. The same expression pattern persists in adult pancreas. Using a genetic model of endocrine pancreas regeneration (mice expressing IFN-γ under the control of the insulin gene promoter), it was proposed that SDF-1 is involved in endocrine cell renewal [34]. More recently, it has been shown that SDF-1 promotes β-cell survival, via activation of Akt, in adult mouse islets [35]. However, it is not known if SDF-1/CXCR4 signaling plays a role in early pancreas development.

In this paper we show that pancreatic epithelial morphogenesis occurs according to a sequence of two opposite epithelial transitions. During the first transition the monolayer of endodermal cells lining the primitive gut gives rise to two pancreatic buds, each composed of a mass of non polarized epithelial cells. During the second transition, this mass reorganizes into branched polarized epithelial monolayers that further differentiate into ducts and acini. Similar sequential transitions were described earlier in the SMG [12,14]. In the pancreas and SMG, *sdf1 *was expressed by the mesenchyme and *cxcr4 *by the epithelial cells. Using embryonic explants of pancreas and SMG, and mice deficient in SDF-1 signaling, we uncovered a new role for SDF-1 in branching morphogenesis.

## Results

### Primitive duct morphogenesis and cell polarization during pancreas development

**Figure 1:**
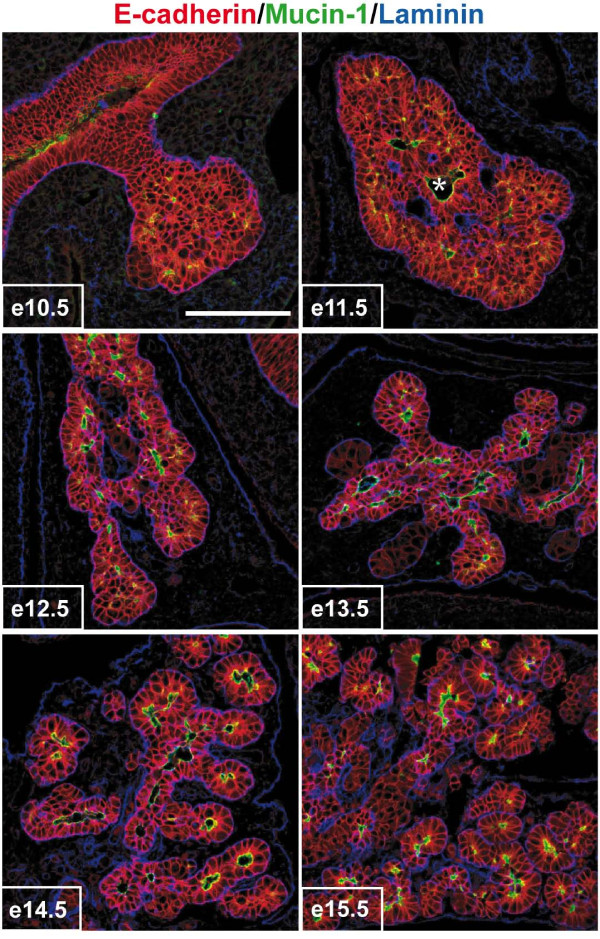
**Remodeling of the pancreatic epithelial cell mass into polarized monolayers**. Pancreatic sections from e10.5 to e15.5 embryos were examined by immunofluorescence using antibodies directed against E-cadherin, mucin-1 and laminin. Pancreatic development starts by the formation of a mass of non-polarized epithelial cells (e10.5-e11.5). This mass is then remodeled (e12.5-e13.5) and finally every epithelial cell become polarized, with its basal pole contacting laminin and its apical pole facing a lumen (e14.5-e15.5). * indicates the central duct, in connection with the duodenum. Scale bar, 100 μm.

To obtain a better insight into the regulation of branching morphogenesis in developing pancreas, we first analyzed primitive duct morphogenesis and epithelial cell polarization. We visualized epithelial cells (E-cadherin), their apical pole lining the lumen (mucin-1), and the basal lamina (laminin) separating the epithelial cells from the mesenchyme on sections from e10.5 to e15.5 pancreas (Figure 1). At e10.5 and e11.5, the pancreatic buds had a globular shape, and most epithelial cells were not polarized, as they were not lined by laminin (basal marker) and did not show mucin-1 (apical marker). At e12.5, the shape of the pancreas became irregular. Lumina of primitive ducts, still surrounded by several layers of epithelial cells became detectable from e13.5 onwards. The pancreas then progressively gave rise to the primitive ductal network, composed of a polarized and monolayered epithelium surrounded by mesenchyme. At e15.5, all epithelial cells were polarized and showed apical, lateral and basal domains (Figure 1). Very few apoptotic cells were detected during these stages, and they were exclusively localized in the non-epithelial compartment (Additional file 1). We concluded that the formation of the primitive pancreatic ductal network occurs by remodeling of a globular mass of epithelial cells, rather than from iterative branching of a monolayer.

**Figure 2:**
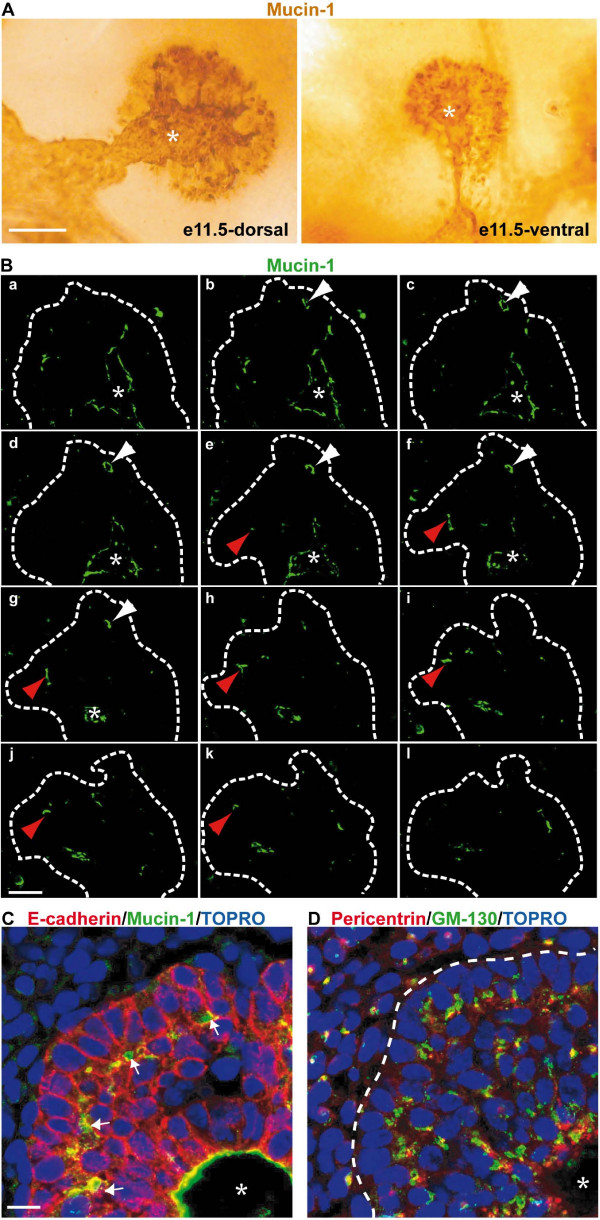
**The pancreatic buds consist of an epithelial cell mass with peripheral lumina**. (A) Whole mount immunoanalyzis of dorsal and ventral pancreatic buds at e11.5 with an anti-mucin-1 antibody. The staining delineates the apical pole of cells lining the lumen of the primitive gut tube, the pancreatic central duct (*) and its extensions. It also shows blind lumina at the periphery of the pancreatic bud. (B) Gallery of pictures from a 3D confocal acquisition on a whole-mount immunofluorescence analyzis of e11.5 dorsal pancreas with anti Mucin-1 (green) antibody. Blind peripheral lumina can be observed from b to g (white arrowheads) and from f to k (red arrowheads). (C, D) Sections of e11.5 dorsal pancreatic bud stained with antibodies against E-cadherin and mucin-1 (C) and pericentrin and GM-130 (D). Nuclei are labeled with TOPRO. Epithelial cells at the periphery of the pancreatic bud (E-cadherin, dashed line) show polarized staining of mucin-1 (arrows), pericentrin and GM-130. * indicates the central duct. Scale bars, 50 μm (A); 20 μm (B); 10 μm (C, D).

We then further characterized the morphology and the cells of the e11.5 pancreatic bud. In addition to the central duct lumen (asterisk in Figure 1, 2A, and Additional file 1), intense mucin-1 stainings also appeared as dots at the periphery of the pancreatic buds (Figure 2A). These stainings corresponded to small developing lumina, called here secondary lumina. Three-dimensional confocal reconstructions were generated to determine the spatial relationship between these secondary lumina and the central duct lumen. No connection between the central and the peripheral lumina were detected, indicating that the latter are blind and independent from the central lumen (Figure 2B; white arrowhead, b to g; red arrowhead, f to k; Additional file 2). Furthermore, a section through the e11.5 pancreatic bud stained for E-cadherin and mucin-1 expression showed that the cells of the outer layer are morphologically different from internal cells (Figure 2C). The outer cells had a cylindrical shape and a basal nucleus. Their apical pole faced the peripheral lumina, as shown by mucin-1 staining (arrows in Figure 2C). In addition, the localization of two intracellular markers in this outer epithelial layer was typical of polarized cells (Figure 2D). The Golgi marker GM-130 was found on the apical side of the nucleus, and the microtubule-organizing center (MTOC) detected by pericentrin staining was located in the subapical region of the cells. In contrast, except for the cells that lined the central duct lumen, the internal cells of the pancreatic bud showed no mucin-1 expression and the localization of the Golgi and MTOC was random. Altogether, these data indicated that the pancreatic bud is a compact epithelial mass, in which the cells are not polarized, except for the outer cells which all have basal and lateral membrane domains; some cells also posses an apical domain.

**Figure 3:**
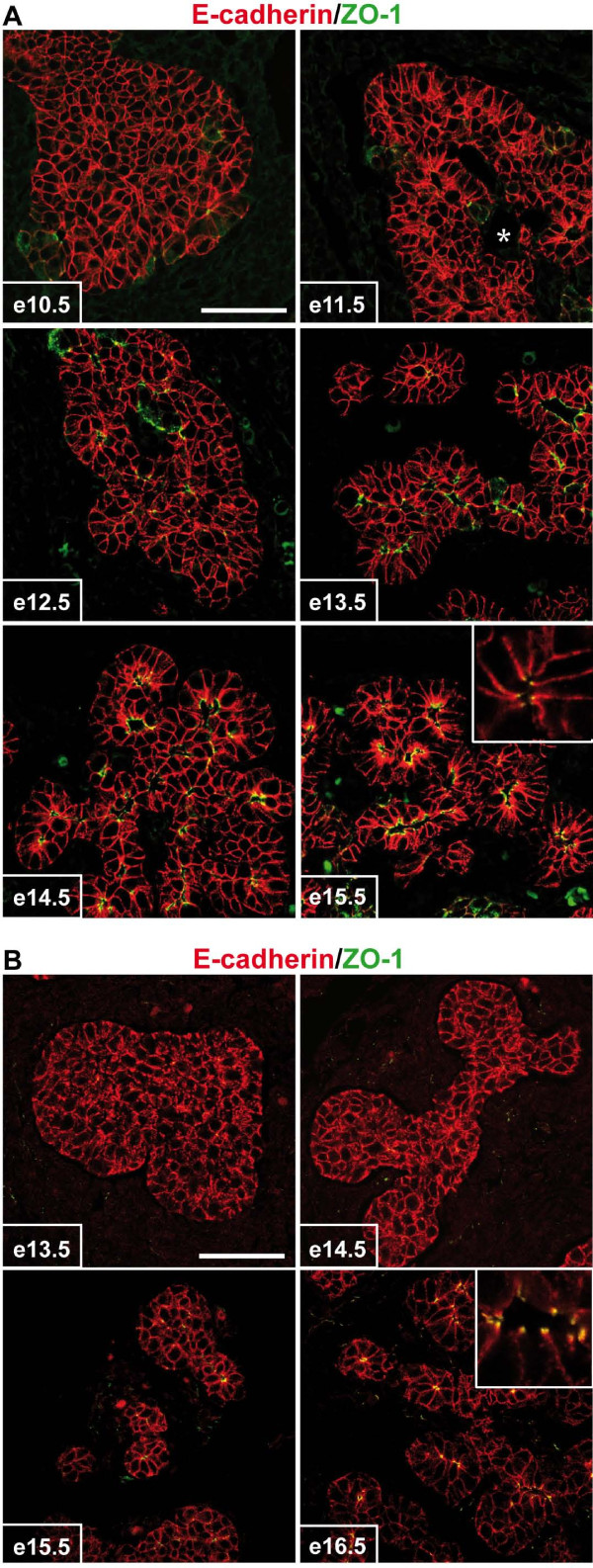
**Primitive duct morphogenesis occurs by transition from epithelial mass to monolayers in the pancreas and in the SMG**. Pancreatic sections from e10.5 to e15.5 (A) and SMG sections from e13.5 to e16.5 (B) embryos were examined by immunofluorescence using antibodies directed against ZO-1 and E-cadherin. Both organs show that duct development occurs by remodeling of a mass of epithelial cells into polarized monolayers. Before remodeling, E-cadherin is expressed all around the cells, whereas it is restricted to the baso-lateral membranes in the monolayered epithelium. In the latter, ZO-1 (tight junction) separates the E-cadherin-negative apical pole from the E-cadherin-positive lateral domains (insets). * indicates the central duct, in connection with the duodenum (A). Scale bars, 50 μm.

To characterize the acquisition of cell polarity during the transition of a globular pancreatic bud to the monolayered epithelium lining the primitive ducts, we analyzed the formation of tight junctions. The latter play a role in cell adhesion and in the establishment and maintenance of apico-basal polarity [36]. To this end, we studied the localization of the tight junction-specific protein ZO-1 from e10.5 to e15.5 (Figure 3A). Despite the continuous presence of adherens junctions (E-cadherin) between pancreatic epithelial cells, strong ZO-1 staining was not detected in the pancreas before e12.5. From that stage on, ZO-1 was progressively seen in cells facing a lumen (Figure 3A). Therefore, ZO-1 localization at the tight junctions was subsequent to the onset of polarization of peripheral cells in the e11.5 pancreatic bud and was concomitant with the remodeling of the epithelial bud into a monolayered epithelium lining the primitive duct lumina.

A similar epithelial transition, from a mass to monolayers, also occurs during submandibular gland (SMG) morphogenesis [12,13]. As shown in Figure 3B, ZO-1 staining was not visible in the early stage of SMG morphogenesis and only became visible after remodeling of the epithelial mass into monolayers lining a lumen (Figure 3B). High magnification pictures of epithelial cell monolayers clearly demonstrated that ZO-1 separates the apical domain (now devoid of E-cadherin staining) from the lateral domain (insets in Figure 3A and 3B).

### SDF-1 and its receptor CXCR4 are expressed in developing pancreas and SMG

**Figure 4:**
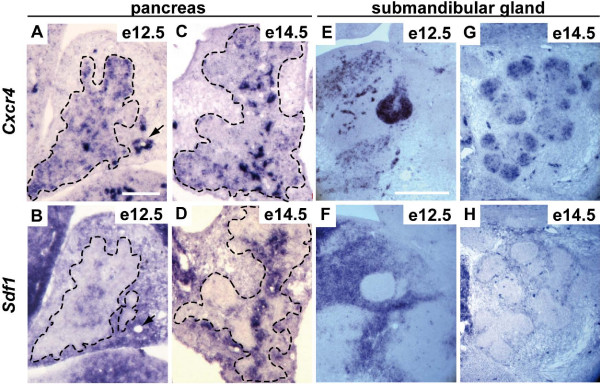
**SDF-1 and its receptor CXCR4 are expressed in developing mouse pancreas and SMG**. Pancreatic (A-D) and SMG (E-H) sections at e12.5 (A, B, E and F) and e14.5 (C, D, G and H) were processed for *in situ *hybridizations using antisense probes for SDF-1 and for CXCR4. SDF-1 is expressed in the mesenchyme and its receptor in epithelial cells (pancreatic epithelium is delineated by a dotted line). At e14.5, SDF-1 staining is also found in pancreatic epithelial cells. Capillaries are positive for both the ligand and receptor (arrows). Scale bars, 100 μm (A-D); 200 μm (E-H).

The mesenchyme is a source of morphogenesis-inducing signals in the pancreas [37] and in the SMG [17]. Therefore, we looked for mesenchymal factors that could regulate epithelial morphogenesis in these organs and considered SDF-1 as a candidate. Indeed, this chemokine and its G protein-coupled receptor CXCR4 have been proposed to control cell movement in many developing organs [38], including the endoderm during zebrafish gastrulation [39]. At the late stages of pancreas development, this signaling pathway is involved in pancreatic cell migration and β-cell survival [34,35]. We first investigated the expression pattern of SDF-1 and CXCR4 in the pancreas by performing *in situ *hybridization experiments. We focused on embryos at e12.5, when epithelial cells are organized in a globular mass, and e14.5, when remodeling has been initiated. We found that *sdf1 *is mainly expressed in the mesenchyme, with lower staining intensity in the epithelium; *cxcr4 *was found in epithelial cells, as early as e12.5 (Figure 4A and 4B). *Sdf1 *and *cxcr4 *expression was also found in the vasculature (arrows in Figure 4A and 4B). This expression pattern was maintained up to e14.5, a stage at which *sdf1 *expression in the central most region of the epithelium became intense (Figure 4C and 4D).

In the developing SMG, the expression of *Sdf1 *and *Cxcr4 *was comparable to that in the pancreas (Figure 4E to 4H). From e12.5 to e14.5: *sdf1 *was expressed in the mesenchyme and *cxcr4 *in the epithelium. Moreover, CXCR7, the second receptor for SDF-1, was observed by immunolocalization in endothelial structures of the SMG (Additional file 3), but not of the pancreas (not shown). From this set of expression data, we concluded that the spatial and temporal expression pattern of SDF-1 and CXCR4 in the pancreas and SMG suggests a potential role for SDF-1 signaling in their development.

### SDF-1 signaling controls pancreatic branching morphogenesis

**Figure 5:**
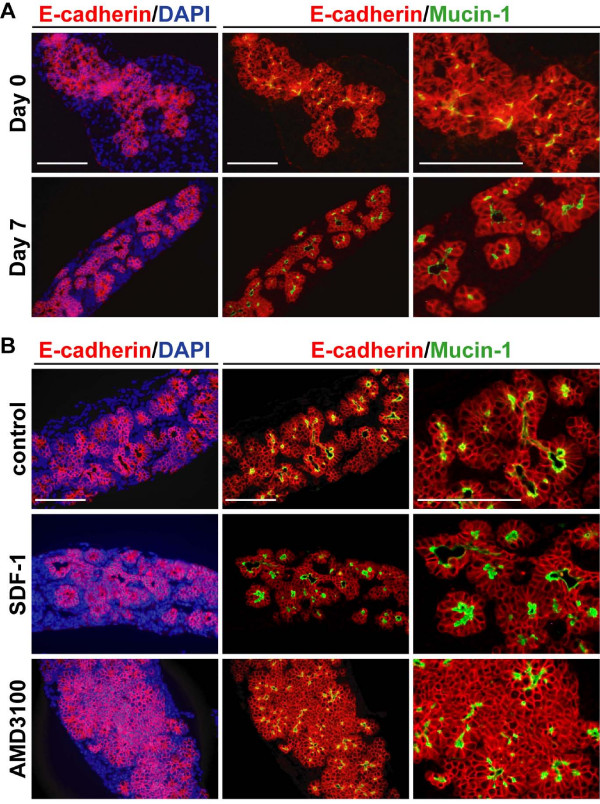
**SDF-1 signaling controls pancreatic branching morphogenesis**. Immunofluorescence analyzis of pancreatic tissue stained for E-cadherin, mucin-1 and DNA. (A) The dorsal pancreatic bud was dissected from e12.5 embryos and either fixed and processed for immunostainings (= e12.5 = day 0) or cultured on filter for 7 days (= day 7) prior to immunostaining analyzis. Dissected mouse e12.5 pancreas before the culture (Day 0) appears as a mass of epithelial cells (red; E-cadherin-positive) surrounded by mesenchyme (E-cadherin-negative). Some cells accumulate mucin-1 at their apical pole, but no lumina are formed. After 7 days in culture (Day 7), the epithelial mass has been remodeled and cells are polarized and form monolayers delineating lumina. (B) e12.5 pancreatic explants dissected from wild-type mouse and cultured for 7 days. Control and SDF-1 (300 ng/ml) treated explants form polarized monolayers delineating lumina and imbricated with mesenchyme. In contrast, treatment of the pancreatic explants with 20 μM AMD3100, a specific pharmacological inhibitor of CXCR4, inhibits epithelial morphogenesis, as seen by the maintenance of the epithelial cells in clusters. Scale bars, 50 μm.

To address the role of SDF-1 signaling in epithelial morphogenesis, we resorted to explants of e12.5 pancreas cultured on filters. Pancreatic cultures have only been validated for analyzing pancreatic cell differentiation when investigating the role of extracellular factors [40]. Therefore, we first validated the pancreatic explants as a model for studying branching epithelial morphogenesis. At the start of the culture (Day 0 in Figure 5A) the explants presented as an epithelial cell mass surrounded by mesenchyme, showing early signs of branching and small, independent, lumina. After 5 to 7 days of culture, the epithelial mass was remodeled into tubulo-acinar epithelial monolayers imbricated within the mesenchyme, as described *in vivo *(Figure 1). Most epithelial cells had acquired polarity with the apical pole facing a well-defined lumen, as revealed by mucin-1 staining, and the basal pole facing the mesenchyme (Day 7 in Figure 5A). We concluded that cultured pancreatic explants provide a model to analyze the formation of the primitive ductal network.

To investigate if SDF-1 controls epithelial morphogenesis in developing pancreas, we performed gain- and loss-of-function experiments by treating cultured explants with exogenous recombinant SDF-1 and with a specific pharmacological inhibitor of CXCR4 (AMD3100) [41]. Pancreatic explants cultured for 7 days were analyzed by immunofluorescence with antibodies against E-cadherin and mucin-1. No effect of exogenous SDF-1 on epithelial organization was observed as compared to control explants (Figure 5B). The absence of effect of exogenous SDF-1 is likely to result from the presence of endogenous SDF-1, whose activity cannot be amplified. In contrast, we found that upon AMD3100 treatment, morphogenesis was impaired (Figure 5B). Epithelial monolayers were not formed and the epithelial cells remained clustered. The mesenchyme was not imbricated with the epithelium and remained at the periphery of the explants. The lumina, as revealed by mucin-1 staining, were smaller and often surrounded by multiple layers of epithelial cells. Identical results were obtained with explants from e11.5 embryos (data not shown), and the effect of AMD3100 was dose-dependent, since milder anomalies were observed at 10 μM than at 20 μM. Thus, at the end of the culture, the AMD3100-treated explants resembled the e12.5 pancreas before the onset of remodeling into a monolayered epithelium (compare AMD3100 panels of Figure 5B with Day 0 panels of Figure 5A). AMD3100 treatment did not change the proliferation or the apoptosis index (Additional file 4A, B) and this was supported by the normal size of these explants. Immunostaining with antibodies against insulin, glucagon or carboxypeptidase-A revealed normal differentiation in the explants treated with AMD3100 (Additional file 4C). From these experiments we concluded that SDF-1/CXCR4 signaling is potentially required for the remodeling of the epithelial cell mass into primitive pancreatic ducts lined by a monolayered epithelium.

### SDF-1 signaling controls SMG branching morphogenesis

**Figure 6:**
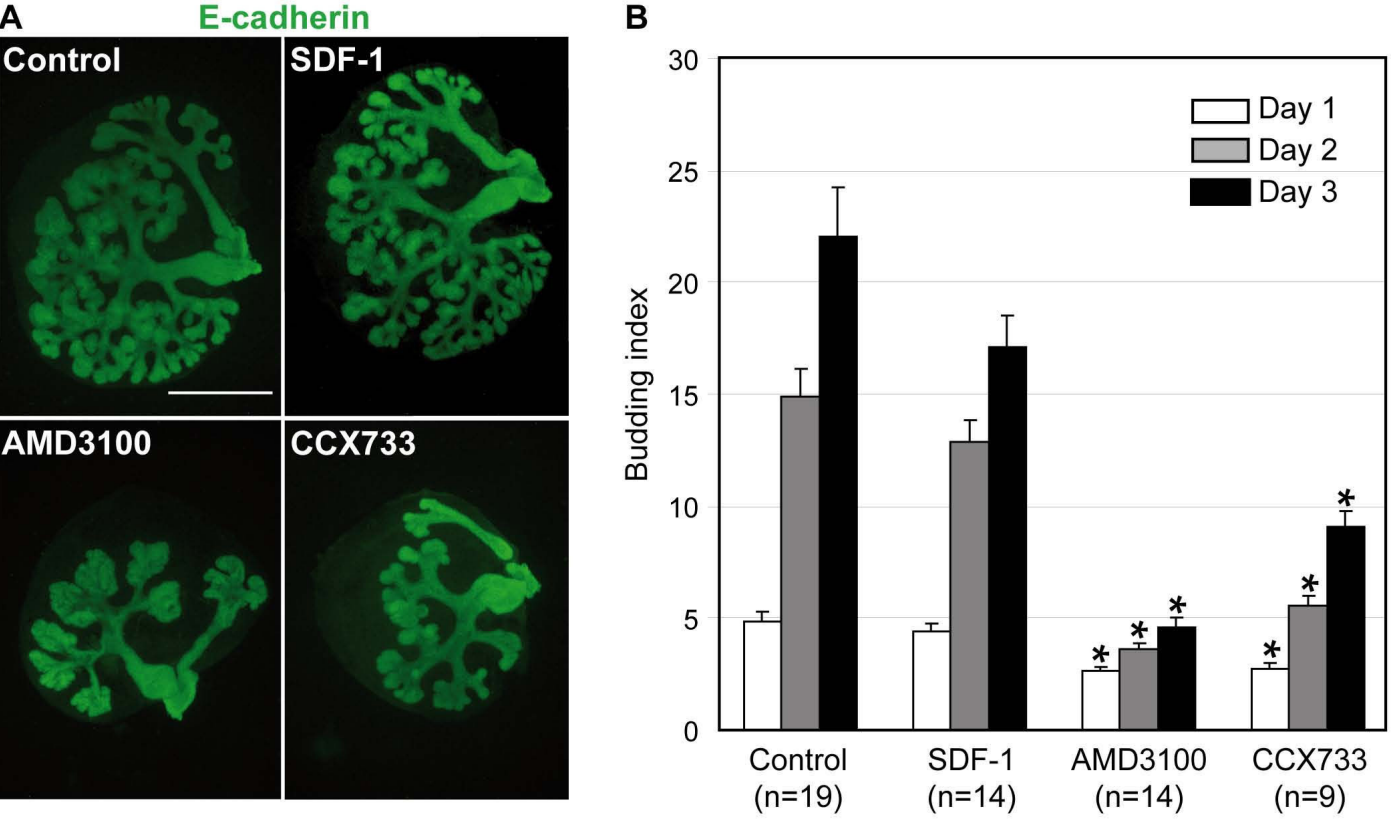
**SDF-1 signaling controls SMG branching morphogenesis**. (A) Representative whole-mount immunostaining of E-cadherin in explants cultured for three days with the control medium, SDF-1, AMD3100 or CCX733. Control and SDF-1-treated cultures show that the epithelium has invaded the whole culture and forms a complex arborescence. In contrast, treatment of the SMG explants with 20 μM AMD3100 inhibits epithelial branching morphogenesis, as seen by the failure to colonize the explant mesenchyme and the reduced number of buds. Similarly, CCX733 reduces the branching as well as the invasion of the mesenchyme. (B) The numbers of terminal buds in each cultured SMG explants were counted at day 0, 1, 2 and 3, and the budding index was calculated by dividing the number of buds at day 1, 2 and 3 by the number of buds at day 0. *t*-test: *, *P *< 0.001. Scale bar, 500 μm.

To investigate if SDF-1 modulates branching morphogenesis of other exocrine glands, we addressed the role of SDF-1 signaling in SMG epithelial morphogenesis. As shown above, this organ develops according to the same two-stage epithelial transition as in the pancreas [12,14,42]. We resorted to cultures of e13.5 embryonic explants, as described earlier by others [14-19]. The SMG epithelial bud surrounded by its mesenchyme was excised and cultured on filter for up to 3 days in the presence of SDF-1 and SDF-1 antagonists. Similarly to the pancreas, gain-of-function experiments with exogenous SDF-1 did not affect SMG branching (Figure 6A). However, upon AMD3100 treatment, morphogenesis of the SMG was impaired (Figure 6A). The size of the glands was smaller and the number of epithelial buds was reduced, as compared to control glands, indicating that SDF-1 signaling is required for branching. Quantification of the number of buds in AMD3100-treated explants revealed a reduction in the budding index (number of buds at day 1, 2 or 3 divided by the number of buds at day 0), as early as one day after the onset of the treatment, and culminating at 80% reduction after 3 days, as compared to control cultures (Figure 6B). As CXCR7 was also expressed in the blood vessels surrounding the SMG (Additional file 3), the SDF-1 antagonist CCX733, which prevents SDF-1 from binding to CXCR7, was also tested. As shown in Figure 6A, CCX733 inhibited SMG morphogenesis by reducing the number of epithelial buds. This was confirmed by a 60% reduction in the budding index as compared to controls after 3 days (Figure 6B). These experiments suggested a role for SDF-1 signaling via CXCR4 and CXCR7 in the branching morphogenesis of the SMG.

**Figure 7:**
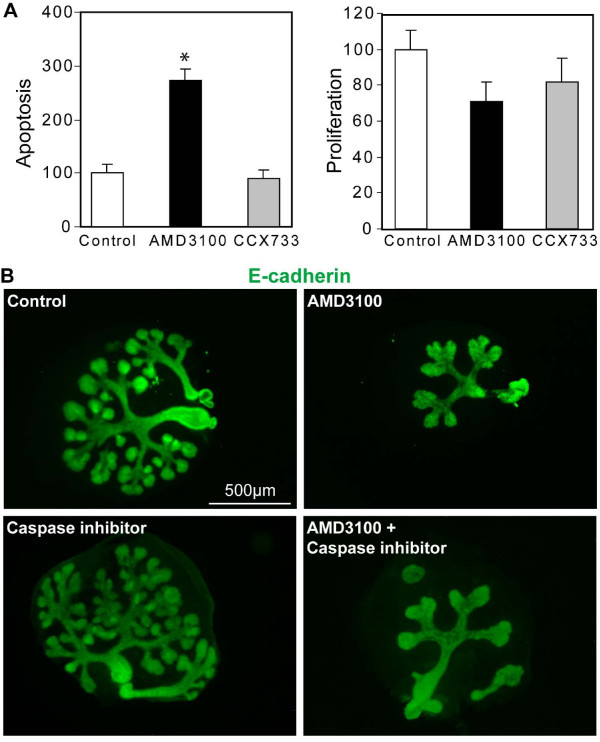
**SDF-1 signaling promotes epithelial cell survival in the SMG**. (A) Quantification of the number of epithelial cells positive for the cleaved caspase 3 (left panel), or phosphohistone H3 (right panel) in SMG explants cultured for 24 hours. All the epithelial cells positive for the marker were counted on every sixth sections of the explant and total number was normalised to its size. Values for control explants were set as 100. *t*-test: *, *P *< 0.05. (B) Representative whole-mount immunostaining of E-cadherin in explants cultured for two days in the presence or absence of a general caspase inhibitor. Blocking caspase activity does not affect branching activity in control or AMD3100-treated explants. Scale bars, 500 μm.

We further investigated the morphogenic defect observed by the addition of AMD3100 to the culture. As SDF-1 signaling can control both cell survival and cell proliferation in different cell types, blocking SDF-1 binding to CXCR4 with AMD3100 could explain the reduced size of the glands. Therefore, we performed immunodetection of phosphohistone H3, a marker of mitosis, and of activated caspase 3, a marker of cell apoptosis, in control, AMD3100- and CCX733-treated explants. Quantification of these stainings revealed that proliferation was not affected when SDF-1 signaling was abrogated (Figure 7A). However, apoptosis showed a three-fold increase in the epithelial buds of AMD3100-treated explants (Figure 7A). This observation could explain the reduced size of the SMG epithelium. We then assessed whether the increase in apoptosis was the cause of the branching defect. For this, we cultured explants in the presence of AMD3100 alone or in combination with a general inhibitor of caspases. Apoptosis was effectively prevented as revealed by the near complete absence of activated caspase 3 staining on sections from caspase inhibitor-treated explants (Additional file 5). However, in the explants co-cultured with AMD3100 and the caspases inhibitor, the branching defect was still observed (Figure 7B), indicating that the reduced branching activity is not a consequence of increased apoptosis. Since SDF-1 did not affect cell survival in developing pancreas (see above) but did control branching morphogenesis of the pancreas, we conclude that SDF-1 has differential effects on organ development, but that it has common effects on epithelial branching.

### Genetic deficiency of CXCR4 or SDF-1 transiently affects epithelial morphogenesis

**Figure 8:**
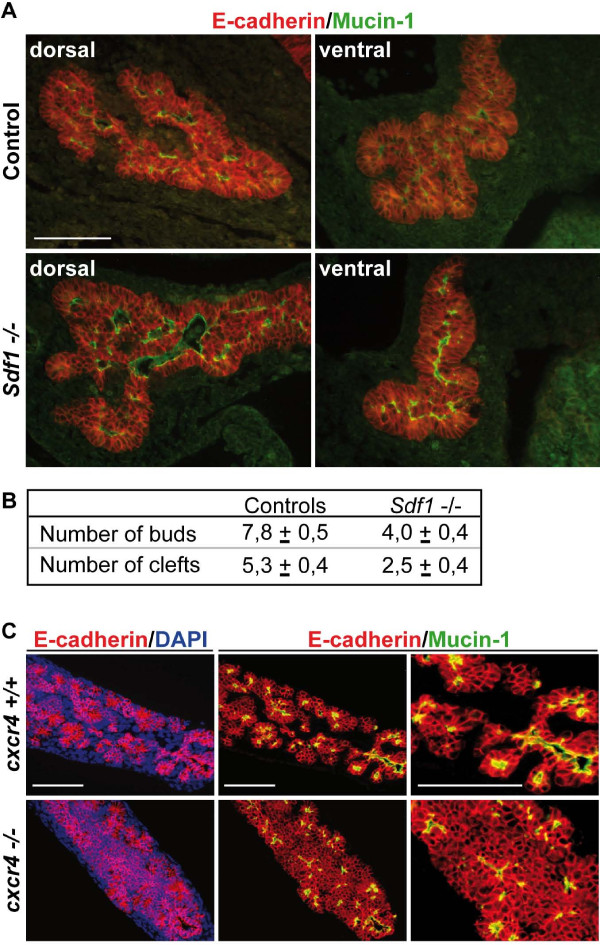
**SDF-1 signaling controls pancreatic branching morphogenesis *in vivo***. (A) Representative immunostainings of two e12.5 control and *sdf1 *knockout pancreata with E-cadherin and mucin-1 antibodies. Reduced branching activity is observed in *sdf1*^-/- ^ventral and dorsal pancreas (B) Quantification of the degree of branching in four e12.5 control and four *sdf1 *knockout pancreata. The organs were sectioned and the number of buds and clefts were counted on every sixth sections and the average numbers per ventral pancreata sections are given. (*P *< 0.0005) (C) Pancreatic explants were dissected at e12.5 from wild-type or *cxcr4*^-/- ^mouse embryos and cultured for 7 days. CXCR4-deficient explants do not remodel into polarized epithelial monolayers and exhibit the same morphogenetic defects as wild-type explants treated with AMD3100. Scale bar, 50 μm.

To verify *in vivo *the relevance of our *ex vivo *observations, we analyzed the morphogenesis of embryonic pancreas in *sdf1 *knockout mice. At e12.5 the ventral pancreas in *sdf1 *knockouts showed less branching and had a more globular shape as compared to controls, confirming the role of SDF-1 in branching morphogenesis (Figure 8A). The degree of branching was evaluated by counting the number of buds and clefts in control and knockout ventral pancreas. This quantification revealed a two-fold decrease in the number of buds in the absence of SDF-1 (Figure 8B). However, the dorsal pancreas did not show obvious branching defects, but was more elongated than in controls and it adopted a hammerhead-like structure while the controls were more rounded. The branching defect was transient since e13.5-e14.5*sdf1*^-/- ^embryos did not show deficient morphogenesis (data not shown).

Since the consequences of *Sdf1 *ablation *in vivo *were rather modest as compared to the effects of *Sdf1 *inhibition *in vitro*, we looked at *cxcr4-/- *pancreas. At e12.5 and e15.5, the dorsal and ventral pancreata were not significantly different in *cxcr4-/- *embryos and in wild-type animals (not shown). However, when cultured on filters *cxcr4*^-/- ^pancreatic explants showed a strong morphogenic deficiency, similar to that seen when wild-type explants were cultured in the presence of AMD3100, a SDF-1 antagonist (compare Figure 8B with Figure 5B). This similarity proves that AMD3100 specifically targets and inhibits SDF-1 signaling via the CXCR4 receptor. Taken together, these data indicated that SDF-1 controls pancreas morphogenesis *in vivo*, but also suggest that compensatory effects operating *in vivo *mask part of the role of SDF-1. We also analyzed the development of SMG in *cxcr4*^-/- ^and *sdf1*^-/- ^embryos, but the SMG phenotypes appeared normal, both *in vitro *and *in vivo*. We concluded that no genetic evidence for a role of SDF-1 signaling in SMG branching could be collected. However, the existence of compensatory mechanisms for genetic deficiencies in the pancreas suggests that compensation may also occur in the SMG.

## Discussion

In this paper we show that the primitive pancreatic ductal network, which is lined by a monolayered and polarized epithelium, forms by remodeling of a globular mass of non polarized epithelial cells. SDF-1 is expressed in the mesenchyme surrounding the epithelium and its receptor CXCR4 is found in the epithelial cells of the pancreas. SDF-1 signaling promotes branching morphogenesis of the pancreas *in vitro *and *in vivo*. We also provide *in vitro *evidence that SMG morphogenesis is controlled by SDF-1.

**Figure 9:**
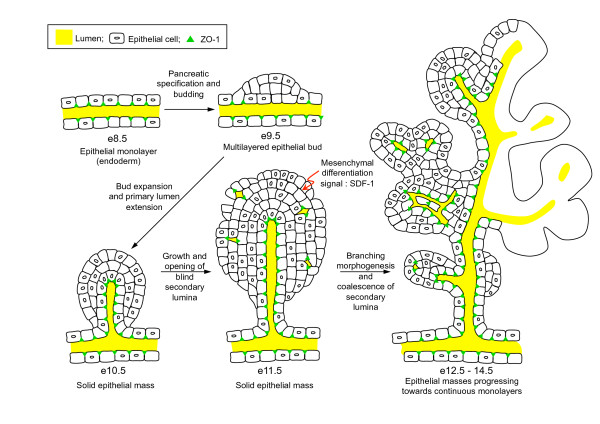
**Development and banching morphogenesis of the pancreas**. Pancreatic specification of polarized endodermal cells leads to the formation of a multilayered and then globular mass of epithelial cells with a central duct lumen (pancreatic bud). This lumen, connected to the gut tube lumen, forms extensions within the pancreatic bud, and is surrounded by several layers of cells, linked by adherens junctions (E-cadherin), but lacking tight junctions (ZO-1). Around e11.5, before remodeling of the mass, some cells at the periphery acquire apico-basal polarity and delineate blind lumina (secondary lumina). Initial branching morphogenesis events result from the coalescence of secondary and central lumina, allowing the progression towards epithelial monolayers.

Previous models depicting pancreatic duct morphogenesis in mammals suggest that it results from the branching of a polarized epithelium [7,43]. We show here that formation of the primitive ductal network is not initiated by iterative branching of a tubular structure extending from the gut (Figure 9). The pancreatic bud at e10.5-e11.5 consists of a globular mass of epithelial cells with a central duct lumen. This lumen, connected to the gut tube lumen, forms extensions within the pancreatic bud, and is surrounded by several layers of cells. These cells are linked by adherens junctions (E-cadherin), but lack tight junctions (ZO-1). At this stage, we observe that some cells at the periphery of the mass show apico-basal polarity and delineate blind lumina (secondary lumina). Based on these observations, we propose that primitive duct branches arise from extensions of the central gut-derived duct that fuse with secondary lumina that appear at e11.5. Interestingly, this process bears resemblance with lumen development in the zebrafish gut tube and exocrine pancreas. Indeed, in these organs the lumen arises by fusion of multiple lumina [44,45]. After the formation of primitive ducts, further branching and expansion of the pancreatic ducts occur by iterative lateral branching, as illustrated by Puri and Hebrok [46]. This is also similar to the development of the mouse SMG, where the epithelial cells first form a mass that becomes remodeled into a monolayered epithelium. Like in the pancreas, epithelial cells in the SMG express E-cadherin and β-catenin. ZO-1 is only detectable when cells are lining a lumen [11,12,42].

The time-course and 3-D analysis of embryonic pancreas development indicated that an initial phase of polarization occurs in cells of the outermost layer of the pancreatic bud at e11.5. This precedes remodeling of the bud, suggesting that, at the onset of pancreas development, remodeling may be triggered by the acquisition of polarity and not the reverse. Beyond this stage, acquisition of apico-basal polarity and primitive duct formation cannot be dissociated morphologically but must be orchestrated by several regulators acting on distinct aspects. Indeed, in our culture explants, blocking SDF-1 signaling did not prevent epithelial cells in the mass to acquire apical characteristics such as assembly of tight junction complexes and primary cilia (Additional file 4 and data not shown). Nevertheless, this polarization program could not go to completion, as the cells remained clustered and most of them did not acquire a basal domain.

During the remodeling of the pancreatic globular mass, the epithelium is in contact with the mesenchyme. The latter tissue has been proposed to provide signals that control epithelial proliferation, differentiation and morphogenesis. We have identified here SDF-1/CXCR4 signaling as a potential regulator of branching morphogenesis. Indeed, SDF-1 and its receptor, CXCR4, are expressed in embryonic pancreas in a complementary fashion. When epithelial cells are still organized as a non polarized mass, SDF-1 is expressed in the mesenchyme whereas CXCR4 is localized in the epithelium. Moreover, when SDF-1 signaling is inhibited by pharmacological means or by CXCR4 genetic deficiency, pancreas morphogenesis is perturbed. These data are consistent with a model in which the mesenchymally-produced SDF-1 promotes epithelial remodeling and thus primitive duct morphogenesis. While this work was under review, Ueland and collaborators demonstrated that SDF-1 controls epithelial kidney morphogenesis in vitro [47]. Our data in the pancreas and SMG, together with those of Ueland et al. demonstrate the role of SDF-1 in epithelial branching morphogenesis. The molecular nature of the branching defects does not involve a change in the proliferation or apoptosis indexes (this study), but may be explained by a change in the migration potential of epithelial cells [47].

There is no perfect overlap between the results obtained with the pharmacological inhibition and with genetic inactivation of SDF-1 signaling in the pancreas. Pharmacological inhibition of SDF-1 signaling *in vitro *severely repressed branching morphogenesis, but genetic deficiency in *Sdf1-/- *pancreas showed more modest effects *in vivo*. Also, the effect of *cxcr4 *gene ablation only led to abnormal pancreas morphogenesis when the organ was cultured *in vitro*. We suggest that compensatory pathways may be active *in vivo *but not *in vitro*. In our culture system, endothelial cells are present and organized around the epithelium like in vivo, the main difference being that there is no blood circulation in the endothelial network in vitro. We suggest that signal(s) coming directly or indirectly from the circulation could participate in the control of epithelial branching morphogenesis of the pancreas and SMG. Such a model would further extend the role of the vasculature in pancreas development, since others previously showed that blood flow through the aorta is required for dorsal pancreas budding from the endoderm [48,49].

In developing SMG, CXCR4 is expressed in the epithelium and SDF-1 in the mesenchyme, like in the pancreas. This suggests that SDF-1 controls branching morphogenesis by a direct mechanism on the epithelium. Importantly, CXCR7 is detected in endothelial cells of the SMG, but not in the pancreas. In SMG explants, we observed a branching defect when SDF-1 binding to CXCR7 was inhibited by CCX733. This suggests that SDF-1 can also indirectly control epithelial branching morphogenesis, via the endothelium. Activation of CXCR7 in the endothelium may induce the production of an unidentified factor which in turn would signal to the epithelium and act in parallel to or amplify the direct SDF-1 effects on epithelial morphogenesis. In the SMG we also noticed a difference between the effects of pharmacological inhibition of SDF-1 and genetic deficiencies induced by inactivation of the *sdf1 *or *cxcr4 *genes. *In vitro *and *in vivo *development of cultured *cxcr4*^-/- ^and *sdf1*^-/- ^SMG was normal, unlike development of AMD3100- or CCX733-treated explants. This suggests the existence of other ligands that could bind CXCR7, or alternative signaling pathways with which SDF-1/CXCR4/7 might interact in this organ. It has been reported that migrating muscle progenitors cells reach the anlage of the tongue in *cxcr4*^-/- ^or *gab1*^-/-^, but not in the double-mutant mice. A crosstalk between G-protein-coupled receptors and tyrosine kinase receptors may contribute to compensatory mechanisms [50].

Finally, when SDF-1 signaling was blocked, the size of the epithelium was reduced in the SMG but not in the pancreas. Although SDF-1 is important for survival of insulin-producing β-cells [35], we did not observe any effect of SDF-1 signaling on cell survival early in pancreas development, even when e12.5 explants were cultured in the absence of serum (data not shown). This indicates that the survival signals are different in the two organs. A role for SDF-1 in cell survival has already been described in several systems [35,51]. We excluded the possibility that the role of SDF-1 in cell survival in SMG coincided with its role in branching morphogenesis, thereby indicating that SDF-1 plays distinct roles in the SMG.

## Conclusions

In conclusion, the present work shows that the formation of primitive ducts in the pancreas depends on remodeling of a globular mass of epithelial cells, as described earlier in SMG. Our data also identify SDF-1 as a factor that may promote the transition from a mass of epithelial cells towards monolayers in the pancreas and SMG. Understanding the mechanisms of such epithelial transition is relevant to carcinogenesis, which is associated with loss of epithelial polarity and formation of a mass of cells that fills the ductal lumen.

## Methods

### Animals

C57BL/6J *cxcr4 *+/- mice were obtained from, and genotyped according to The Jackson Laboratory (Bar Harbor, MA). The generation of SDF-1-/- mice has been described [28]. All other mice were of the CD1 strain. The animals were raised and treated according to the principles of laboratory animal care of the University Animal Welfare Committee.

### Dissection and culture of explants

Pancreatic and SMG explants were microdissected from mouse embryos at e12.5 and e13.5 respectively and cultured on microporous membranes [40]. M199 medium was supplemented with 10% fetal calf serum for the pancreas and Universal complement Insulin/Transferrin/Selenite (BD Biosciences) for SMG culture. The medium of pancreatic cultures was changed every day. Recombinant mouse SDF-1a (R&D systems, Lille, France) was dissolved in PBS containing 0.15% bovine serum albumin (BSA) to 14 mg/ml (stock solution) and was added at the final concentration of 300 ng/ml. AMD3100 (Sigma, Bornem, Belgium) was dissolved in water to 10 mM (stock solution) and was added at 20 μM in the culture medium. CCX733, in DMSO, was used at 20 μM [27]. The general caspase inhibitor VI (Calbiochem/VWR, Leuven, Belgium) was dissolved in DMSO to 16.5 mM (stock solution) and was added at the final concentration of 25 μM. Control explants were exposed to the same concentration of vehicle as the test samples.

### *In situ *hybridization

Mouse embryos were fixed overnight at 4°C in 60% ethanol, 30% formaldehyde and 10% acetic acid before paraffin embedding. Full-length cDNAs of SDF-1 and of CXCR4 were generated via RT-PCR using as template reverse transcribed total RNA derived from mouse embryo heads. DIG-labeled antisense RNA probes were produced by in vitro transcription of the SDF-1 and CXCR4 cDNAs, and hybridizations were performed on 16 μm-thick sections as described [52].

### Immunodetection

For whole mount immunolocalization, pancreata dissected from embryos were fixed in 4% paraformaldehyde in PBS for 2 h at 4°C and treated as described [53]. For immunofluorescence on sections, embryos or explants were fixed, embedded and processed as described [54]. Antibodies and dilutions used in this study are given in Additional file 6. Sections were stained with bis-benzimide (Sigma, Bornem, Belgium) or with TOPRO in PBS during incubation with the secondary antibodies. Fluorescence was observed with a Zeiss Axiovert 200 inverted fluorescence microscope or with a Biorad confocal microscope or with a Zeiss LSM510 multiphoton confocal microscope.

## List of abbreviations

The abbreviations used are CXCR4: chemokine CXC-motif receptor 4; e: embryonic day; SDF-1: Stromal cell-derived factor-1; SMG: Submandibular glands.

## Authors' contributions

ACH, JVE and CEP planned and carried the dissection, cultures and immunofluorescence experiments, SC and CEP and CF performed the in situ hybridization, LP and HK collected the CXCR4 and SDF-1 embryos, TN, PV, PJC, GGR, FPL and CEP conceived and coordinated the experiments and drafted the manuscript. All authors read and approved the manuscript.

## Supplementary Material

Additional file 1 **Apoptosis does not explain remodeling of the epithelial cell mass**. Pancreatic sections from e10.5 to e15.5 embryos were analyzed by TUNEL assay, and costained using E-cadherin antibody and DAPI. Very few apoptotic cells are observed during epithelial remodeling. * indicates the central duct, in connection with the duodenum. Scale bar, 50 μm. Click here for file

Additional file 2 **Absence of connection between the central and the peripheral lumina**. Movie obtained from approximately 50 images spanning 25 micrometers of a whole-mount immunolabeled e11.5 dorsal pancreas (anti-E-cadherin (red), anti Mucin-1 (green)) using a LSM510 multiphoton microscope. Click here for file

Additional file 3 **CXR7 is expressed in blood vessels of the SMG**. Sections from SMG were examined by immunofluorescence using antibodies directed against E-cadherin, or PECAM (green) and CXCR7 (red). CXCR7 staining is observed in elongated structure positive for the endothelial marker, PECAM. SMG epithelium is delineated by a dotted line. Scale bar, 50 μm. Click here for file

Additional file 4 **AMD3100-treatment does not affect cell proliferation, apoptosis, differentiation and polarization in pancreatic explants**. (A) Pancreatic explants were cultured for 3 days with or without 20 μM AMD3100. Sections were stained with antibodies directed against E-cadherin and phosphohistone H3, a marker of proliferating cells. The localization of the proliferating cells is random. Double-stained cells on 12 sections of two controls and three AMD3100-treated explants were counted. AMD3100 has no influence on the number or the localization of proliferating cells. (B) Pancreatic explants were cultured for 2 days with or without 20 μM AMD3100. Sections were stained with antibodies directed against E-cadherin and cleaved caspase 3, a marker of apoptotic cells. Double-stained cells on 6-7 sections of four controls and 5 to 10 sections of four AMD3100-treated explants were counted. AMD3100 has no influence on the number of apoptotic cells. (C) Immunofluorescence analyzis of pancreatic tissue stained for insulin, E-cadherin or ZO-1 (red) together with glucagon, carboxypeptidase A (CPA) and E-cadherin, respectively (green). e12.5 pancreatic explants dissected from wild-type mouse and cultured for 7 days without treatment (upper panels) or with 20 μM AMD3100 (lower panels). Treatment does not affect the formation of endocrine cell clusters, the expression of pancreatic hormones and exocrine enzyme, or the formation of tight junctions. Click here for file

Additional file 5 **Increased apoptosis in AMD3100-treated explants is prevented by a general caspase inhibitor**. Immunofluorescence analyzis of explants stained for E-cadherin and cleaved caspase 3. Explants were cultured for two days in the presence of AMD3100 alone or in combination with a general caspase inhibitor. Blocking caspase activity prevents activation of caspase. Scale bar, 100 μm. Click here for file

Additional file 6 List of antibodies used. Click here for file
