# Supplementary figures and images for: Mechanism of primitive duct formation in the pancreas and submandibular glands: a role for SDF-1
Source: BMC Dev Biol. 2009 Dec 14;9:66. doi: 10.1186/1471-213X-9-66 (PMC2801489; doi:10.1186/1471-213X-9-66)

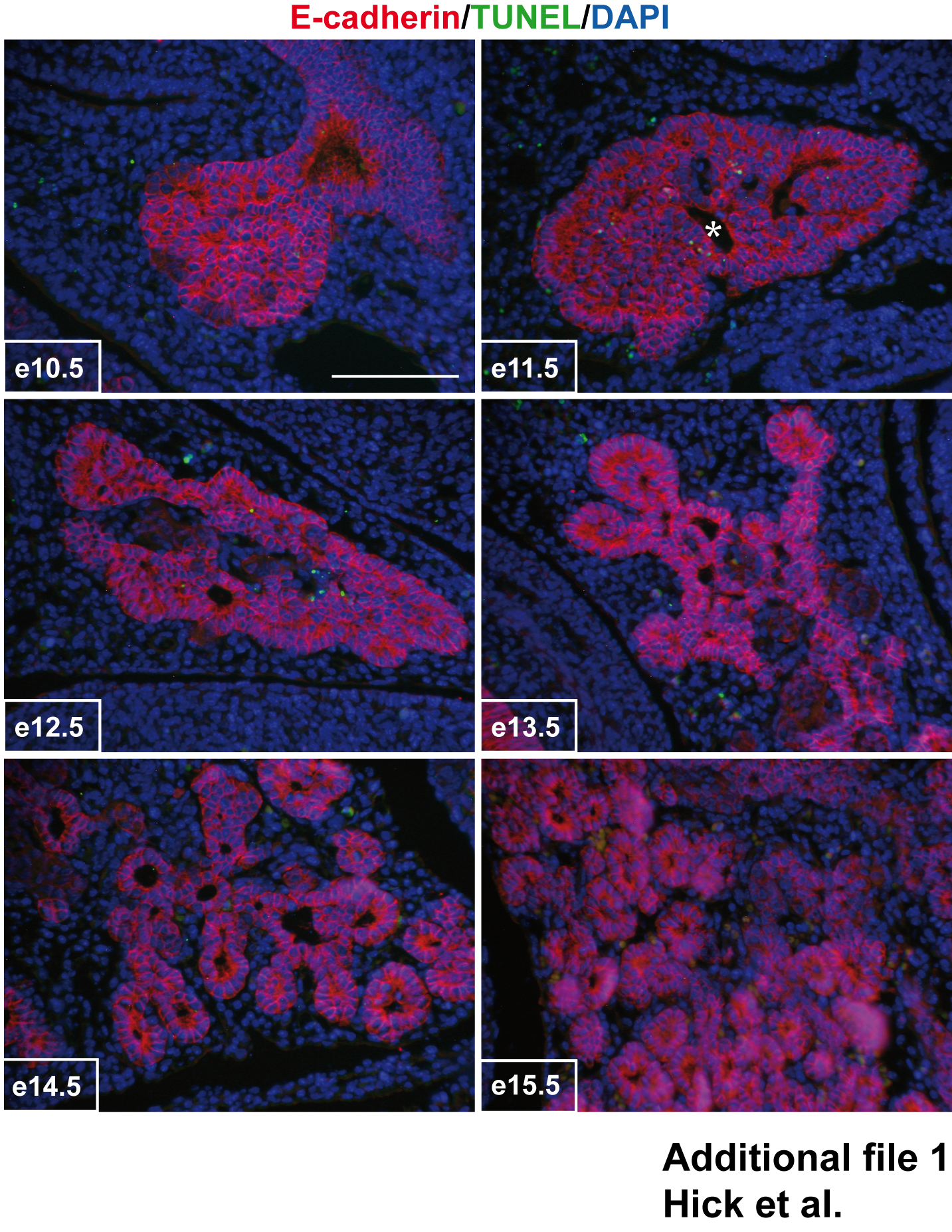

Supplement: Additional file 1 — Apoptosis does not explain remodeling of the epithelial cell mass. Pancreatic sections from e10.5 to e15.5 embryos were analyzed by TUNEL assay, and costained using E-cadherin antibody and DAPI. Very few apoptotic cells are observed during epithelial remodeling. * indicates the central duct, in connection with the duodenum. Scale bar, 50 μm. [file 1471-213X-9-66-S1.PNG]

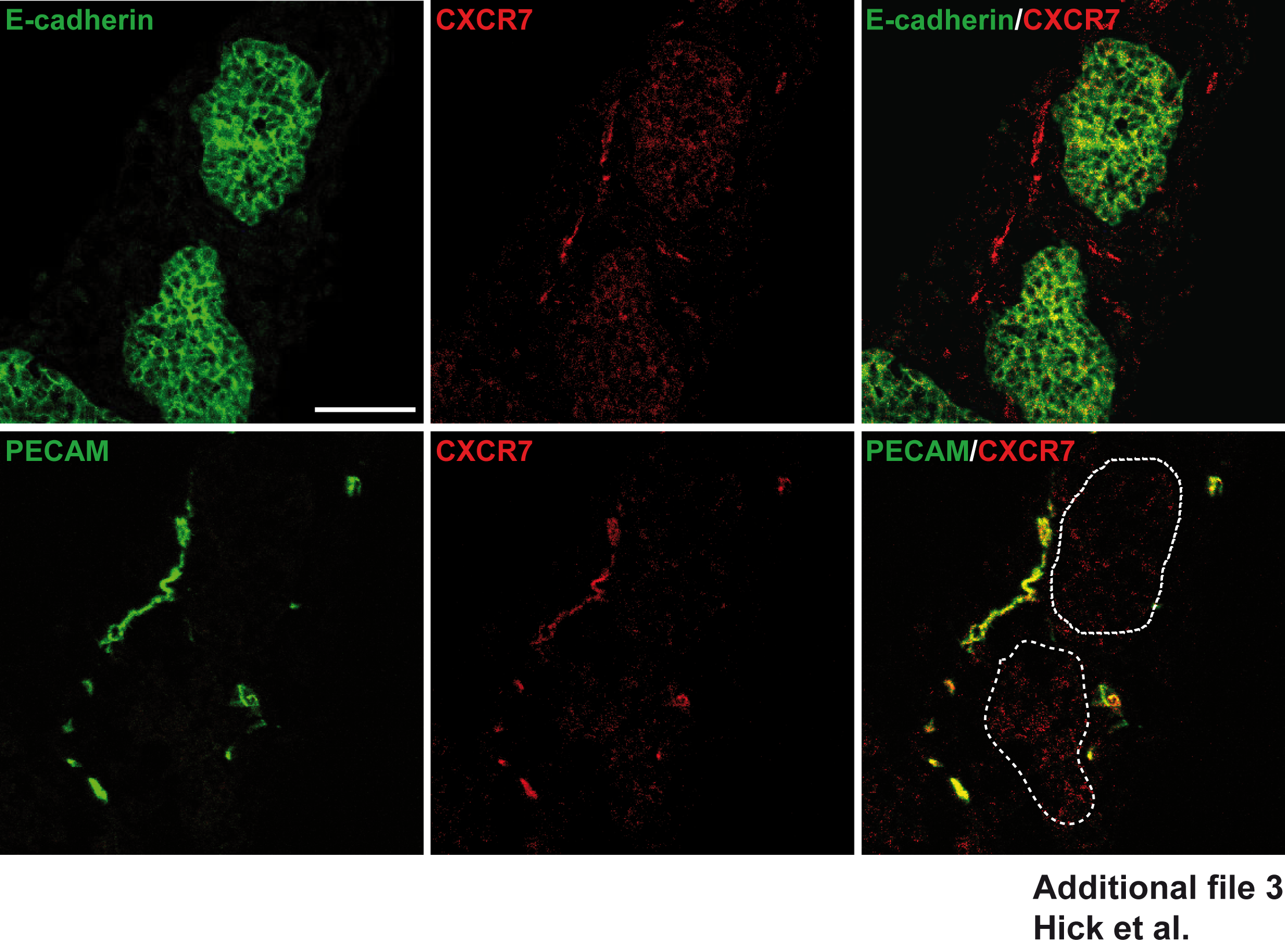

Supplement: Additional file 3 — CXR7 is expressed in blood vessels of the SMG. Sections from SMG were examined by immunofluorescence using antibodies directed against E-cadherin, or PECAM (green) and CXCR7 (red). CXCR7 staining is observed in elongated structure positive for the endothelial marker, PECAM. SMG epithelium is delineated by a dotted line. Scale bar, 50 μm. [file 1471-213X-9-66-S3.PNG]

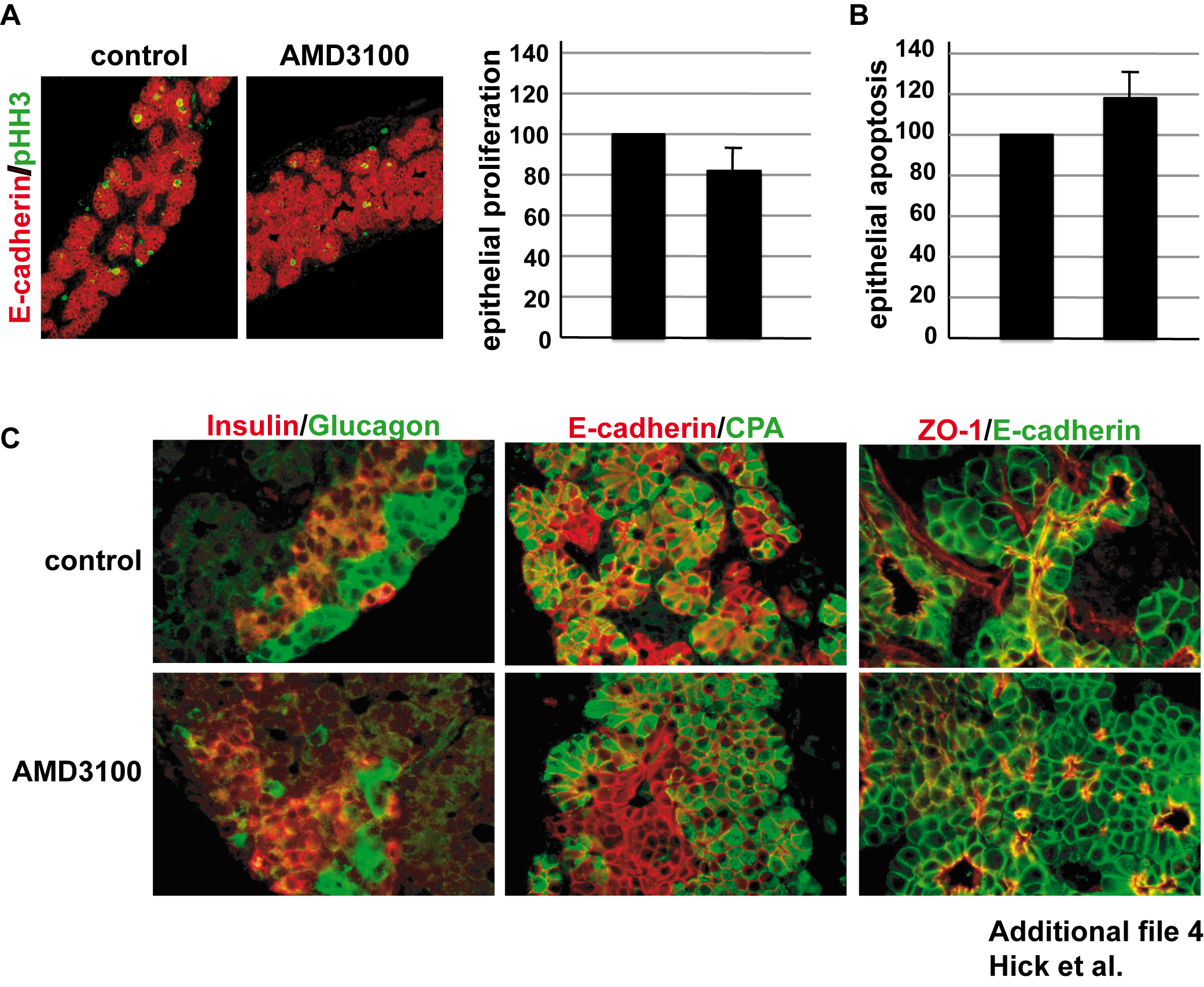

Supplement: Additional file 4 — AMD3100-treatment does not affect cell proliferation, apoptosis, differentiation and polarization in pancreatic explants. (A) Pancreatic explants were cultured for 3 days with or without 20 μM AMD3100. Sections were stained with antibodies directed against E-cadherin and phosphohistone H3, a marker of proliferating cells. The localization of the proliferating cells is random. Double-stained cells on 12 sections of two controls and three AMD3100-treated explants were counted. AMD3100 has no influence on the number or the localization of proliferating cells. (B) Pancreatic explants were cultured for 2 days with or without 20 μM AMD3100. Sections were stained with antibodies directed against E-cadherin and cleaved caspase 3, a marker of apoptotic cells. Double-stained cells on 6-7 sections of four controls and 5 to 10 sections of four AMD3100-treated explants were counted. AMD3100 has no influence on the number of apoptotic cells. (C) Immunofluorescence analyzis of pancreatic tissue stained for insulin, E-cadherin or ZO-1 (red) together with glucagon, carboxypeptidase A (CPA) and E-cadherin, respectively (green). e12.5 pancreatic explants dissected from wild-type mouse and cultured for 7 days without treatment (upper panels) or with 20 μM AMD3100 (lower panels). Treatment does not affect the formation of endocrine cell clusters, the expression of pancreatic hormones and exocrine enzyme, or the formation of tight junctions. [file 1471-213X-9-66-S4.PNG]

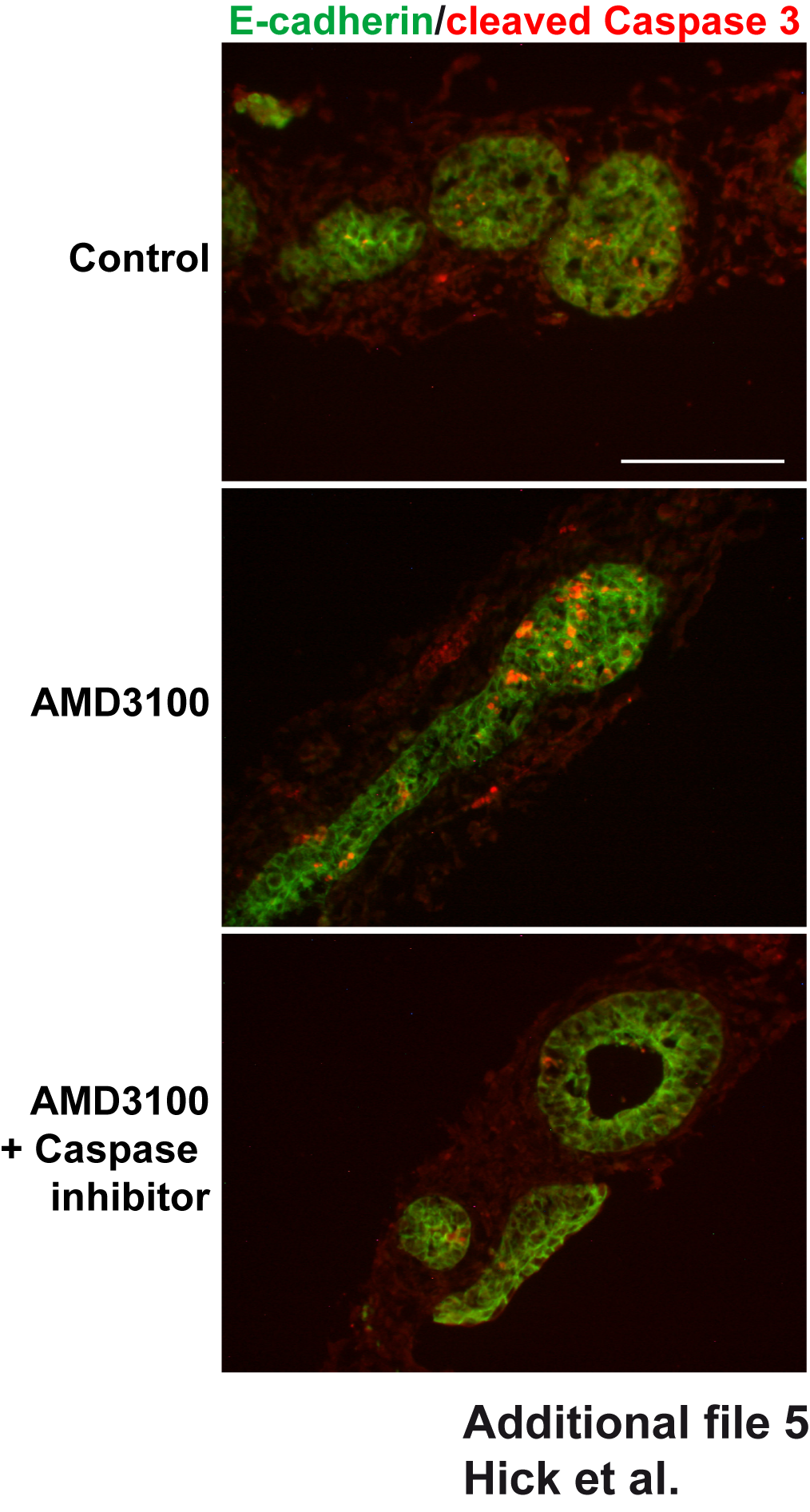

Supplement: Additional file 5 — Increased apoptosis in AMD3100-treated explants is prevented by a general caspase inhibitor. Immunofluorescence analyzis of explants stained for E-cadherin and cleaved caspase 3. Explants were cultured for two days in the presence of AMD3100 alone or in combination with a general caspase inhibitor. Blocking caspase activity prevents activation of caspase. Scale bar, 100 μm. [file 1471-213X-9-66-S5.PNG]
