# Supplementary material for: Mechanism of primitive duct formation in the pancreas and submandibular glands: a role for SDF-1
Source: BMC Dev Biol. 2009 Dec 14;9:66. doi: 10.1186/1471-213X-9-66 (PMC2801489; doi:10.1186/1471-213X-9-66)
Supplement: Additional file 6 — List of antibodies used. [file 1471-213X-9-66-S6.PDF]

Additional file 6: list of antibodies used for immunostaining experiments

primary antibody	species	dilution	source	catalog number
E-cadherin	mouse	1/1000	BD Biosciences	610182
Muc1	hamster	1/200	Neomarkers	HM-1630-P
Laminin	rabbit	1/50	Sigma	L9393
ZO-1	rabbit	1/100	Zymed	61-7300
GM-130	mouse	1/100	BD Biosciences	610822
Pericentrin	rabbit	1/2000	Covance	PRB-432C
CXCR7	mouse	1/400	Chemocentryx	N/A
Insulin	guinea pig	1/100	DAKO-cytomation	A0564
Glucagon	rabbit	1/100	DAKO-cytomation	A-0565
Carboxypeptidase A	rabbit	1/1000	Biogenesis	1810-0006
Acetylated tubulin	mouse	1/5000	Sigma	T6793
Cleaved Caspase 3	rabbit	1/200	Cell signaling	9661
Phopho Histone H3	rabbit	1/50	Cell signaling	9701
PECAM	rat	1/100	BD Biosciences	550274

Secondary molecules and fluorescent probes	species	dilution	source	catalog number
anti-goat/Alexafluor 594	donkey	1/2000	Invitrogen	A11058
anti-mouse/Alexafluor 488	donkey	1/1000	Invitrogen	A21202
anti-mouse/Alexafluor 488 IgG2a	goat	1/1000	Invitrogen	A21131
anti-mouse/Alexafluor 594	donkey	1/1000	Invitrogen	A21203
anti-mouse/Alexafluor 594 IgG1	goat	1/1000	Invitrogen	A21125
anti-rabbit/Alexafluor 594	donkey	1/2500	Invitrogen	A21207
anti-rabbit/Alexafluor 660	goat	1/1000	Invitrogen	A21074
anti-rat/Alexafluor 488	goat	1/1000	Invitrogen	A21208
anti-mouse/Alexafluor 568 IgG1	goat	1/1000	Invitrogen	A21124
strepatvidin/Alexafluor 488		1/2000	Invitrogen	S11223
anti-Armenian Hamster/biotinylated	goat	1/500	Jackson	127-065-160
